# Channel Formation and Membrane Deformation via Sterol-Aided Polymorphism of Amphidinol 3

**DOI:** 10.1038/s41598-017-11135-x

**Published:** 2017-09-07

**Authors:** Masayuki Iwamoto, Ayumi Sumino, Eri Shimada, Masanao Kinoshita, Nobuaki Matsumori, Shigetoshi Oiki

**Affiliations:** 1 Department of Molecular Physiology and Biophysics, Faculty of Medical Sciences, University of Fukui, Fukui, 910-1193 Japan; 2 PRESTO, Japan Science and Technology Agency (JST), Saitama, 332-0012 Japan; 3 Department of Chemistry, Graduate School of Sciences, Kyushu University, Fukuoka, 819-0395 Japan; 4 High-speed AFM for Biological Application Unit, Institute for Frontier Science Initiative, Kanazawa University, Kanazawa, 920-1192 Japan; 5 Bio-AFM frontier Research Center, Kanazawa University, Kanazawa, 920-1192 Japan

## Abstract

Amphidinol 3 (AM3) is an anti-fungal polyene extracted from a marine dinoflagellate. Here, we examined the ion channel activity and membrane-embedded structure of AM3 using a lipid bilayer method and atomic force microscopy (AFM). AM3 exhibited large-conductance (~1 nS) and non-selective single-channel activity only when sterols were present in the membrane leaflet of the AM3-added side. The variable conductance suggests the formation of a multimeric barrel-stave pore. At high AM3 concentrations, giant-conductance “jumbo” channels (~40 nS) emerged. AFM revealed a thicker raft-like membrane phase with the appearance of a wrinkled surface, in which phase pores (diameter: ~10 nm) were observed. The flip-flop of ergosterol occurred only after the appearance of the jumbo channel, indicating that the jumbo channel induced a continuity between the outer and inner leaflets of the membrane: a feature characteristic of toroidal-like pores. Thus, AM3 forms different types of sterol-aided polymorphic channels in a concentration dependent manner.

## Introduction

**Figure 1:**
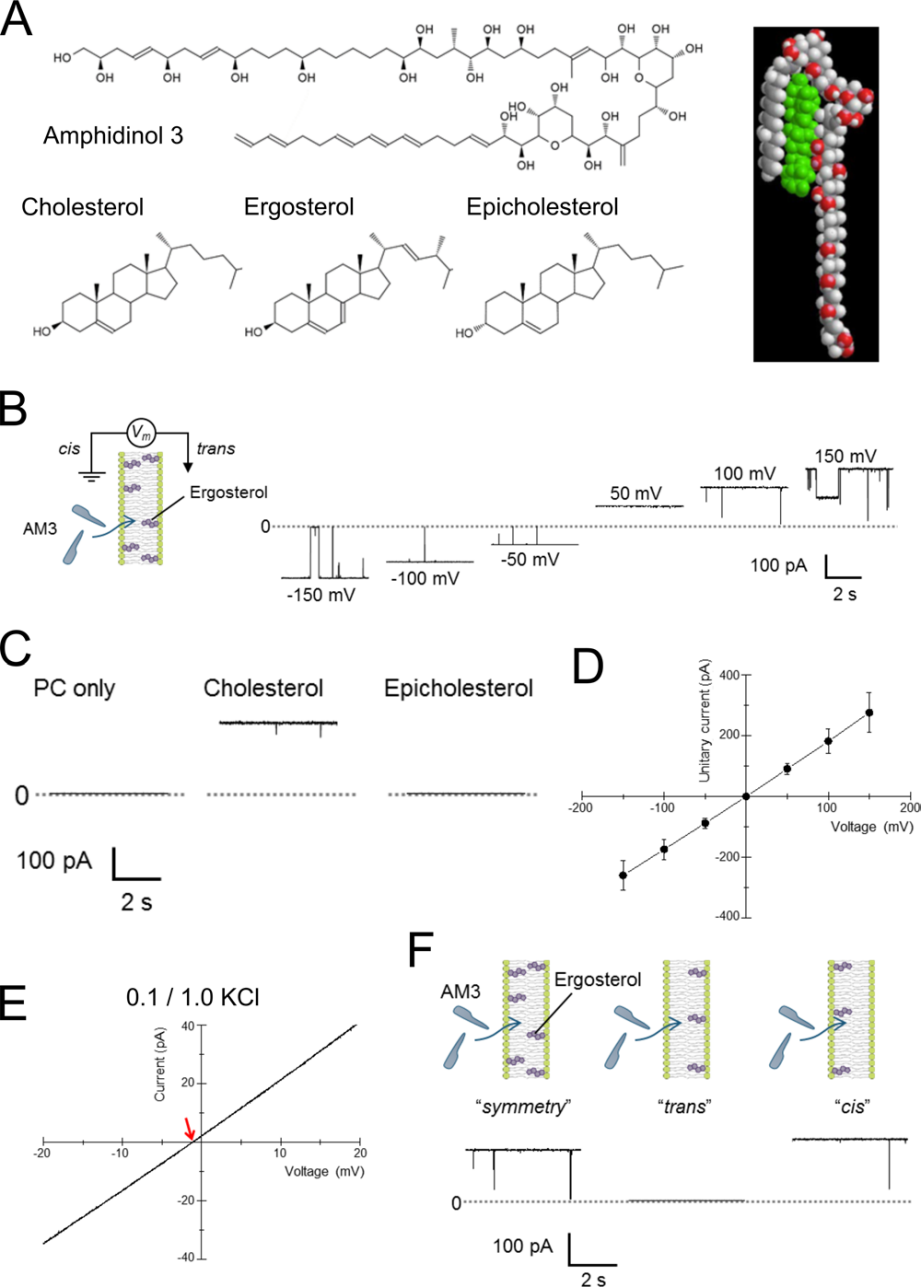
Sterol-dependent formation of the AM3 channel. (**A**) Chemical structures of AM3 and sterols. Epicholesterol is an epimeric form of cholesterol. In cholesterol, the hydroxyl group is in the *β*-conformation, whereas in epicholesterol, the hydroxyl group is in the *α*-conformation. A model of the interaction between AM3 and ergosterol is also shown (green structure denotes an ergosterol molecule). (**B**) Single-channel current recordings of the AM3 channel. The experimental conventions, such as the side of AM3 addition and the definition of membrane potential, are shown in an inset. Representative current traces at different membrane potentials are shown. The composition of the lipid bilayer was 90 mol% POPC and 10 mol% ergosterol. The membrane was formed using the contact bubble bilayer method. AM3 (20 nM) was added to the *cis* compartment (1.0 M KCl = the *trans* compartment), which was grounded for current recordings. The channel showed discrete gating behavior, and a sub-conductance level was observed. (**C**) Sterol dependency of channel activity. (**D**) Single-channel current-voltage curve of the AM3 channel in the ergosterol-containing membrane. (**E**) Reversal potential obtained from the single-channel current-voltage curve in asymmetric solution. The KCl concentration of the *cis* side was 0.1 M, and that of the *trans* (working electrode) side was 1.0 M. The reversal potential was −1.59 mV, indicating slight cation selectivity. (**F**) Sidedness of the ergosterol effect on channel activity. Asymmetric membranes were formed by the contact bubble bilayer method (see Methods). Channel activity was observed when ergosterol (purple) was present in the *cis* side of the membrane. The concentration of AM3 was 20 nM, and that of KCl was 1.0 M.

Amphidinol 3 (AM3)^1, 2^ is a novel polyene extracted from the marine dinoflagellate *Amphidinium klebsii* that possesses anti-fungal and hemolytic activities. Its cytotoxic effect is mediated by the formation of a large pore in the target cell membrane, which was elucidated by the analysis of calcein leakage^3–5^. Similar to other polyene macrolides, such as nystatin and amphotericin B^6–8^, sterols are necessary for pore formation of AM3^4, 5^. In these polyene macrolides, ion channels are formed through the assembly of oligomers with the aid of sterols in the membrane. The sterol specificity of channel formation affords an opportunity to select target cells^9^. In AM3, the stereospecificity of sterols for pore formation has been elucidated^5^. However, unlike other polyene macrolides, AM3 has a chemical structure that is linear rather than macrocyclic (Fig. 1A): an acyl chain and a long polyene chain are connected to two tetrahydropyran rings^1, 2^. Thus, the mechanism of pore formation for other polyene macrolides in the membrane^10^ is not applicable for AM3.

Here, we characterized the channel activity of AM3 using a lipid bilayer method^11^ and its membrane-embedded structure using atomic force microscopy (AFM)^12–14^ at the single-channel level. We found that AM3, with its unique structure, exhibited unprecedented and wide-spectrum features of channel activity. Moreover, at a high AM3 concentration, phase separation^15^ occurred in the AM3-embedded membrane, similar to sterol-containing rafts^16, 17^. The unique features of AM3, including its polymorphic channel activities and ability to induce membrane rafts, were discussed in relation to the toxic actions in the targeted cells as well as an advanced tool for membrane manipulation.

## Results

### Sterol-induced channel activity of AM3

We first examined the single-channel activity of AM3 in the ergosterol-containing membrane. Lipid bilayers containing phosphatidylcholine (PC) and ergosterol (PC : ergosterol = 9 : 1; Fig. 1A) were formed using the contact bubble bilayer (CBB) method^11^, and the two aqueous compartments contained buffered KCl solution. AM3 was added to one of the compartment, which side was termed *cis* (Fig. 1B, see Methods). Electrophysiologically, the *cis* side was set as the reference against which the membrane potential was defined for the opposite *trans* side (Fig. 1B scheme; Methods). This definition corresponds to the membrane potential of the cells attacked by AM3 from the extracellular side. A membrane potential of ±200 mV was applied until the channel activities appeared spontaneously, and was then changed to desired membrane potentials for the current recordings. AM3 rarely inserted into the membrane when the membrane potential was lower, suggesting that the membrane thinning induced by electrostriction^18–20^ is one reason for the facilitated insertion of AM3. In the absence of ergosterol or in the pure PC membrane, no channel activities were observed (Fig. 1C).

The single-channel conductance was in the range of 1.7 nS, and a little variation in the conductance suggests that the channels are formed through self-assembled oligomers of AM3 molecules (Fig. 1B,D). The channel remained open most of the time, but closings towards the zero-current level were often observed (Fig. 1B). The conductance was constant before and after the closing events, indicating that conformational changes of a defined oligomeric structure underlie the gating. The single-channel current-voltage curves were almost linear (Fig. 1D). For such large-conductance channels with nS conductance values, the pore diameter is readily estimated according to the macroscopic principle of ion flow^21^; the rough estimate of the diameter was ~0.8 nm (see Methods). Ion selectivity of the channel was examined under bi-ionic conditions^22^ (see Methods). As shown in Fig. 1E, the reversal potential was −1.59 mV in 1 M/0.1 M KCl solution, and the permeability coefficient of Cl^−^ over K^+^ was 0.93. Thus, the AM3 channel is practically a non-selective channel.

### Sterol-specific action

When cholesterol was present in the membrane, similar channel activity was recorded (Fig. 1C). There were some distinct differences observed in the channel activity of cholesterol-containing membranes. In contrast to the nearly uniform conductance in ergosterol-containing membranes, the single-channel conductance in cholesterol-containing membranes was more variable (Fig. S1), supporting the formation of channels with variable oligomeric size. Additionally, more frequent gating was observed. The single-channel recording revealed these specific differences in the actions between ergosterol and cholesterol that have not been described in previous studies. Epicholesterol is an OH stereoisomer of cholesterol (Fig. 1A). When epicholesterol was present in the membrane, AM3 did not induce channel activity (Fig. 1C). Thus, the stereospecific interaction between AM3 and sterols^5^ (either ergosterol or cholesterol) is a prerequisite for channel formation.

### Sidedness of sterol action

To elucidate the sidedness of the effect of sterol in the membrane relative to the AM3 insertion side, asymmetric membranes^23^ containing sterol in one of the leaflets were formed (Fig. 1F; see Methods). The membrane leaflet facing the *cis* compartment was called the *cis* leaflet. As shown in Fig. 1F, channel activity was observed only when ergosterol was present in the *cis* leaflet. The same was true for cholesterol. No channel activity was detected even in long recordings in the ergosterol-containing membrane in the *trans* leaflet, indicating that ergosterol is unlikely to flop^24^. These results conform to the proposed complex of AM3 and sterol (Fig. 1A inset), which form self-assembled complexes as a barrel-stave type pore^25–27^.

### Structure of the AM3 channel observed by AFM

**Figure 2:**
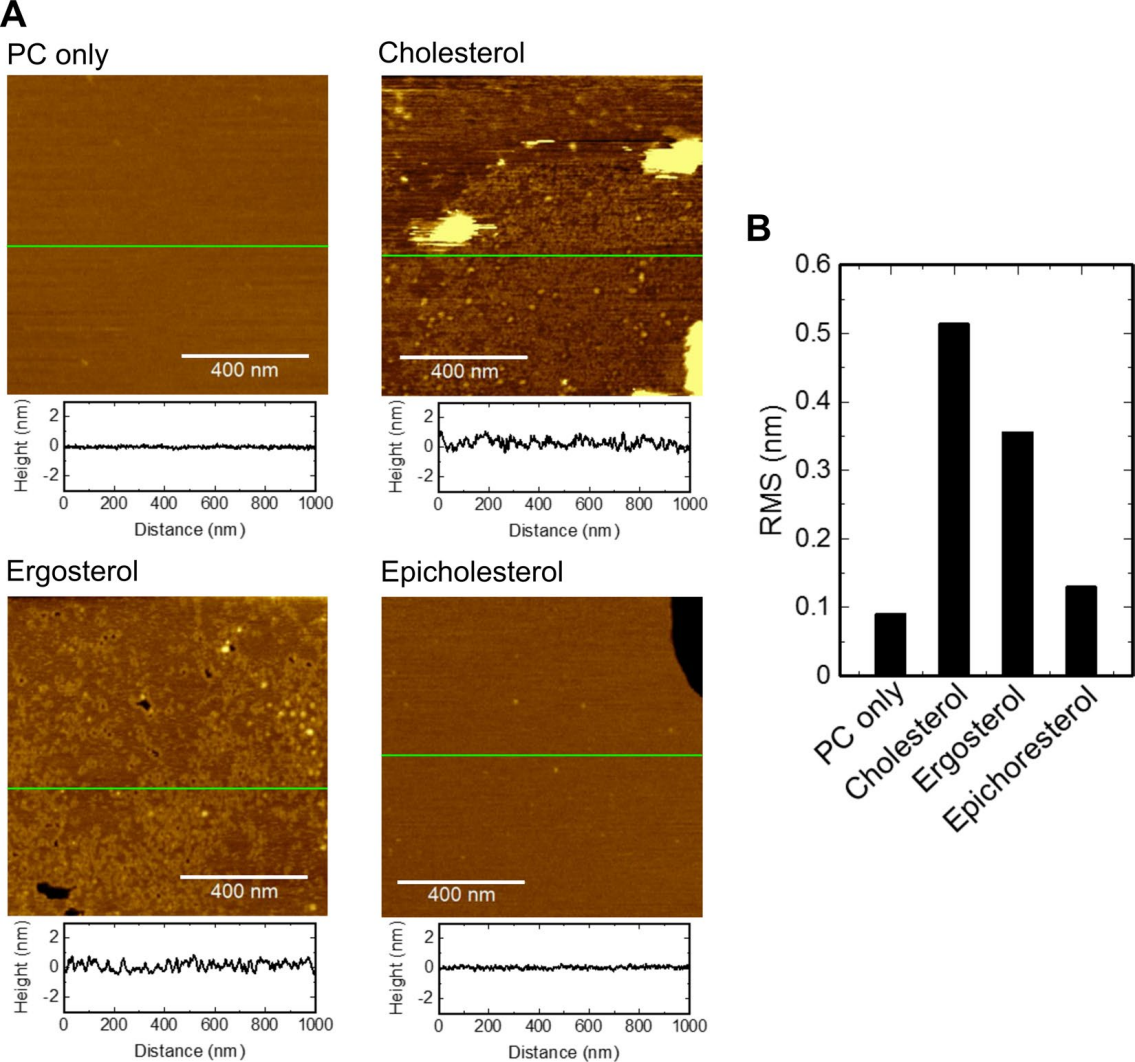
AFM images of the AM3-treated membrane with and without sterols. (**A**) AFM images of the membrane surface. Supported lipid bilayers of PC with and without 10% sterols (cholesterol, ergosterol or epicholesterol) are shown. The concentration of AM3 added to the aqueous solution was 4.5 μM. The height profiles along the green solid lines are shown below the image. The height of the bilayer surfaces was set to 0 nm, and membrane roughness was evaluated. Images were taken in a solution containing 300 mM KCl and 10 mM HEPES (pH 7.5). (**B**) RMS values of the height irregularities of the AFM images in (**A**). Wrinkles are observed in ergosterol- and cholesterol-containing membranes.

The structure of the AM3 channel was examined using AFM. The sterol-containing bilayer membrane was formed on a flat mica surface, and a high concentration of AM3 was added to the aqueous phase (4.5 μM; see Methods for details). Figure 2A shows the surfaces of the membranes. Compared to the control condition without sterol, the membrane surface with ergosterol or cholesterol shows phase separation, having a coarse-grained phase in an otherwise flat membrane phase. With epicholesterol, AM3 did not induce roughness in the membrane. Thus, surface deformation is the result of AM3-sterol (ergosterol or cholesterol) interactions. The roughness of the surface, evaluated as the root-mean-square (RMS) value (Fig. 2B), was larger for the ergosterol- or cholesterol-containing membrane than that without sterol or with epicholesterol.

**Figure 3:**
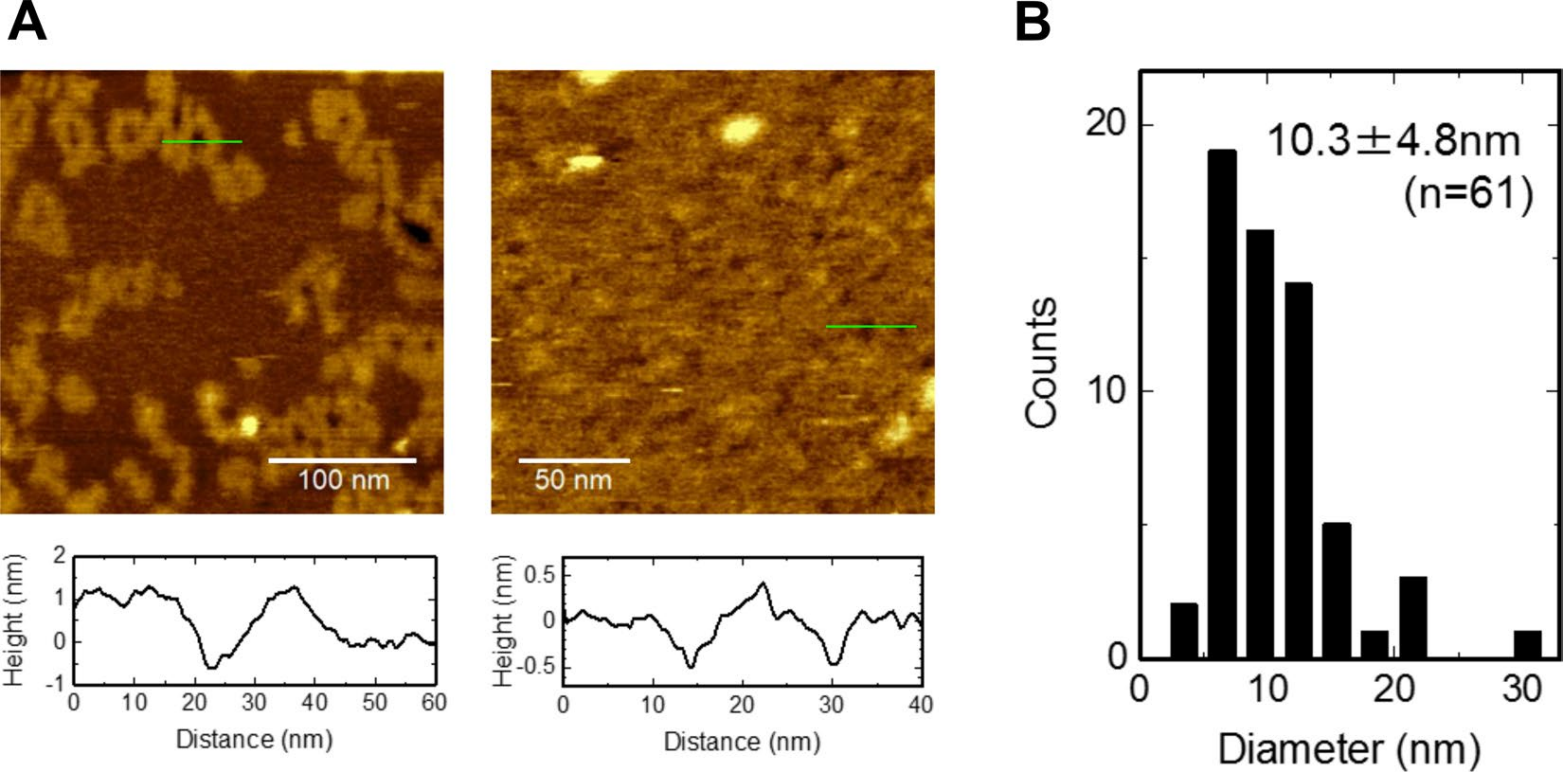
AM3 pores in ergosterol-containing membranes. (**A**) AFM images of AM3 pores. The experimental conditions are the same (4.5 μM AM3, 300 mM KCl, and 10 mM HEPES, pH 7.5) as those described in Fig. 3. Height profiles along the white solid lines are indicated below the images. The height of the bilayer surfaces was set to 0 nm. In the left image, the isolated pores are surrounded by a thick rim, and the ridge without the pore extends tortuously. In the right image, pores are embedded in the coarse-grained phase. (**B**) Histogram of the pore size observed by AFM (n = 61). The diameter of the pores was measured at the level of the membrane surface (at 0 nm height).

In the coarse-grained phase, wrinkles with a defined width were observed. The thickness of the flat surface was 4.7 nm, and the height of the wrinkle from the flat membrane surface was ~0.8 nm (Fig. 3A), while the width of the wrinkle was ~10 nm. More importantly, we detected pores with a diameter of ~10 nm (Fig. 3B). The height profile of the pores demonstrated access of the AFM tip in the pore. However, the inner diameter of the pore, which determines the conductance, remains elusive, because of the limited access of the tip to the narrowest part of the pore. Thus, the ~10 nm diameter is somehow overestimated. Some of the pores were buried in the wrinkled phase, while others were observed on the flat surface with a rim or cuff around the pore. The height and width of the rim around the pore were similar to those of the wrinkles, suggesting that the membrane deformation and pore formation originated from a common process.

**Figure 4:**
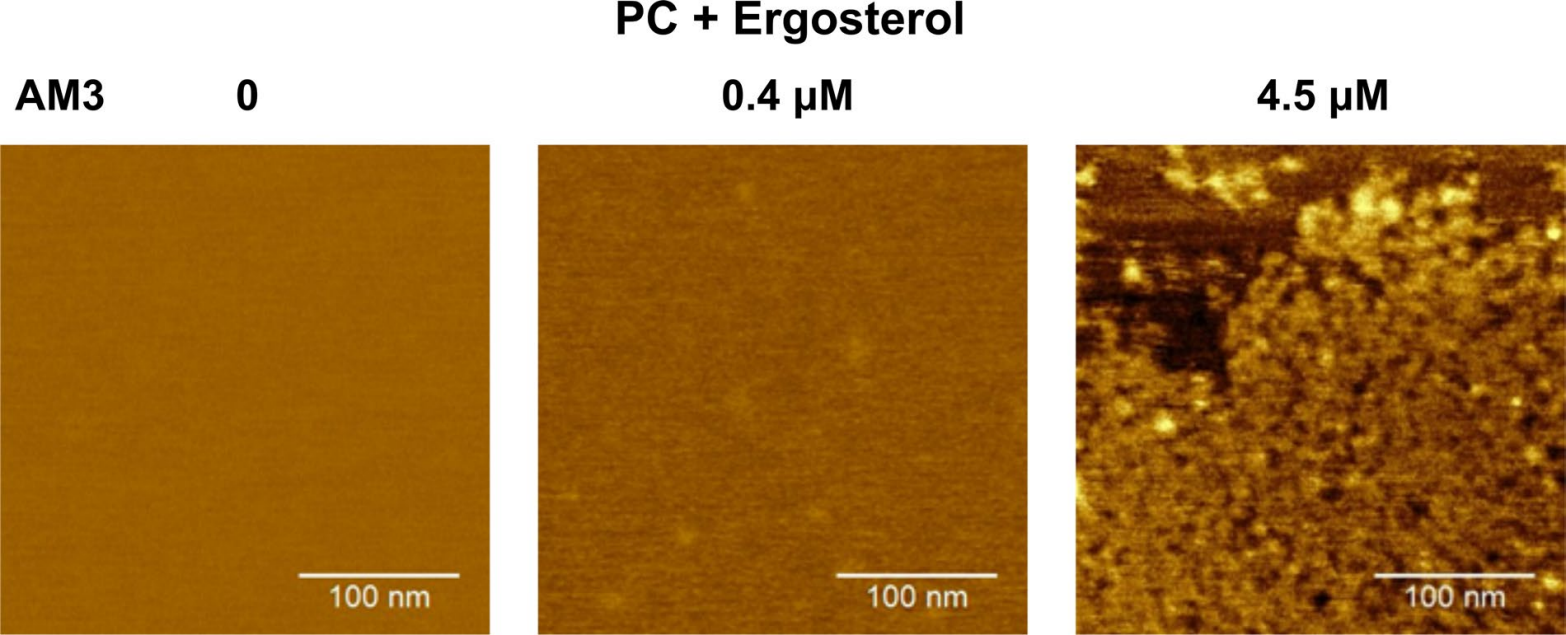
AFM images of the ergosterol-containing membrane at different concentration of AM3. Supported lipid bilayers of POPC with 10% ergosterol are shown. Images were taken in a solution containing 300 mM KCl for 0 and 0.4 μM AM3, and the relevant image at 4.5 μM AM3 was already shown in Fig. 3A. Here, the AFM image in 1000 mM KCl (pH 7.5 with 10 mM HEPES) is shown, which is indistinguishable from that in 300 mM KCl.

AFM images at different AM3 concentrations are shown in Fig. 4. At 0.4 μM, the surface image was distinct from that of the control condition without AM3 (see also Fig. S2 for cholesterol-containing membrane). The surface was mostly flat, and low-profile spots were observed, even though they were sparse. Compared to the coarse-grained surface at 4.5 μM, the pore structure was not resolved, indicating that the 10 nm-large pores do not exist at 0.4 μM. This AFM image at 4.5 μM was obtained in the 1000 mM KCl solution, similar to the electrophysiological recording﻿. The surfaces at 300 mM (Fig. 3A) ﻿and 1000 mM﻿ KCl were compared, but they were  indistinguishable, indicating that the ionic strength does not affect the surface deformation.

In parallel to the AFM imaging, physical nature of the AM3 treated giant unilamelar membrane (GUV) was examined using fluorescence labeled PC (Fig. S3)^28^. The phase separation was not resolved, but the diffusion of PC was progressively decreased as AM3 concentration was increased (Fig. S3). This result indicates that the membrane fluidity was attenuated in the presence of AM3.

### Concentration dependency of the macroscopic current

**Figure 5:**
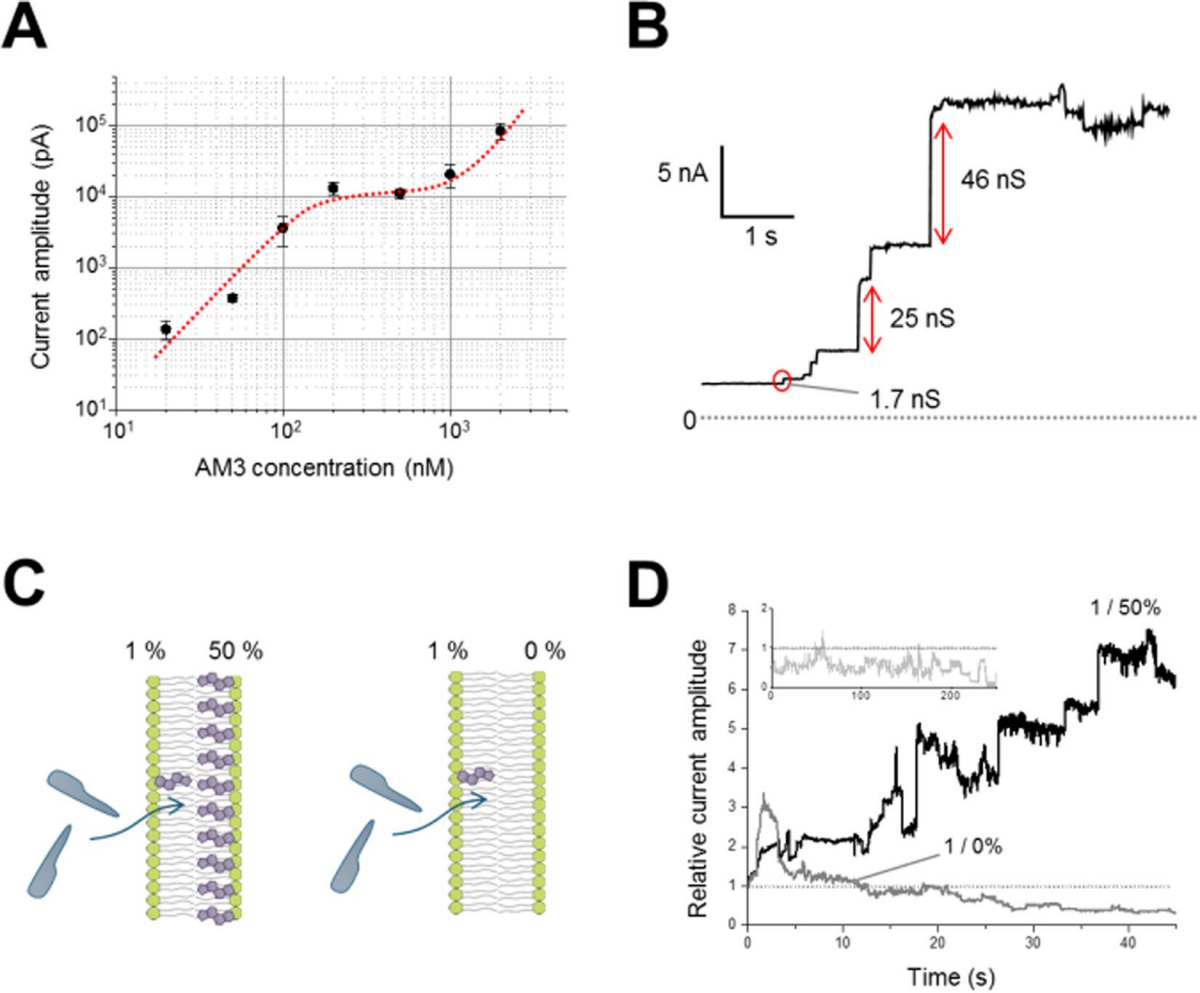
Jumbo channels and time courses of macroscopic current amplitude after the appearance of jumbo channels. (**A**) Macroscopic current amplitude as a function of AM3 concentration. Multiple-phase behavior of channel activity is observed. The fitted line does not have physical meaning. The current was measured at +100 mV in the presence of 10% ergosterol in the membrane at 1 M KCl. (**B**) Channel current recordings of jumbo channels at a high AM3 concentration (2 μM). Jumbo channels appeared spontaneously in the macroscopic activities of small-conductance (1.7 nS) channels. (**C**) Experimental procedure for the *trans*-side effect of ergosterol. The channel activities of AM3 at a high concentration were recorded with a low concentration (1%) of ergosterol in the *cis* leaflet. The concentration of *trans* ergosterol was set as high (50%; left) or zero (right). (**D**) Time course of the ensemble macroscopic current after the appearance of a jumbo channel (AM3 concentration: 1000 nM). Current traces from different membranes were collected after the first appearance of a jumbo channel, and the traces were ensemble-averaged (n = 5). The macroscopic current gradually increased when the ergosterol concentration of the *trans* leaflet was high (black) but decreased in the absence of ergosterol in the *trans* leaflet (dark gray). After the first appearance, several jumbo channels appeared subsequently. At a low AM3 concentration (100 nM; light gray in the inset), the current amplitude was stable throughout the recording time, even though the concentration of the *trans* ergosterol was high.

The pore size measured from the AFM images was much larger than the estimated size obtained by single-channel conductance, while the AM3 concentration in these experiments differed substantially. To integrate these results, the concentration dependence of the AM3 current was examined. The macroscopic current amplitude was saturated at the mid-concentration range (0.5 μM), but increased further at the high-concentration range (2 μM) (Fig. 5A). Channel currents at higher concentrations of AM3 (several μM) could not be attained because the membrane became unstable. This multi-phase behavior in concentration dependency has never been observed for channel-forming substances^6, 29^. What is the underlying mechanism?

### Jumbo channels at high AM3 concentrations facilitate the “flop” of ergosterol across leaflets

Channel activity was examined at a high (μM) AM3 concentration, where membrane instability was circumvented by reducing the ergosterol concentration (1 mol%) in the PC membrane. We found surprisingly jumbo channels as large as 40 nS appeared spontaneously on the background macroscopic current consisting of many low-conductance channels (Fig. 5B). These jumbo channels, having varying conductance (20–50 nS), remained open. The large conductance of the jumbo channels was not due to simultaneous openings of smaller channels, as observed in the high-resolution current recordings. The emergence of jumbo channels was infrequent, and after a few jumbo channels appeared the current amplitude gradually saturated. These features indicate that the distinct type channels from those at low concentration appeared exclusively at high concentration.

From the single-channel conductance, the inner diameter of the pore was estimated to be 4 nm for the 46 nS jumbo channel (see Methods, Eq. 1), which is sufficiently large for the passage of calcein^4, 5^. The size is consistent with the pore size observed in the AFM images: The pore size is overestimated in the AFM image because of the limited access of the tip, while that is underestimated in the current amplitude because of the assumed cylindrical shape.

The jumbo channels have never been observed at the mid-concentration range, suggesting that single-channel conductance does not continually increase as the AM3 concentration increases. Rather, the distinct jumbo type of AM3 channels is formed exclusively at the high-concentration range, accounting for the multi-phase behavior of the concentration-dependent macroscopic current amplitude.

To distinguish functional features of the jumbo channels, an asymmetric membrane^23^ was formed with a low concentration of ergosterol in the *cis* leaflet (1%) and a high concentration in the *trans* leaflet (50%) (Fig. 5C). The addition of AM3 to the *cis* side (2000 nM) generated macroscopic channel currents that involved many low-conductance channels, and the current amplitude remained stable as long as the jumbo channels did not appear. After the emergence of a jumbo channel, the macroscopic current amplitude increased steadily. This behavior is shown in Fig. 5D. The black trace is the ensemble trace of several current traces by aligning the zero time of the first appearance of a jumbo channel. This steady increase suggests that the high concentration of *trans* ergosterol, which is free from AM3 binding, flopped to the *cis* leaflet only when jumbo channels appeared. Then, the flopped ergosterols bound to AM3 on the *cis* side, leading to the formation of channels with increased macroscopic current amplitude. In contrast, when ergosterol was absent in the *trans* leaflet, the macroscopic current began to decrease gradually after the appearance of a jumbo channel at time zero (gray trace). The ergosterol in the *cis* leaflet flipped towards the ergosterol-free *trans* leaflet along the concentration gradient, and this decrease in ergosterol concentration in the *cis* leaflet resulted in the decline in the macroscopic current amplitude. When the AM3 concentration was low (100 nM), the macroscopic current amplitude remained stable even during a long recording time (Fig. 5D inset; light gray), indicating that the flip-flop of ergosterol did not occur. Only the jumbo channel, which appeared at a high AM3 concentration, mediated the flip-flop of ergosterol.

## Discussion

In the ergosterol- (or cholesterol-) containing membrane, AM3 forms large-conductance, non-selective channels that are polymorphic in nature at different concentration ranges. First, the single-channel current recorded at a low AM3 concentration (20 nM) showed individual channel activity with discrete open-closed transitions. The stereospecific action of sterols in the *cis* leaflet indicates that AM3 and sterol form a complex that is self-assembled to form the channel. The conductance was not uniform (approximately 2 nS) in ergosterol-containing membranes, and the conductance variability was higher in cholesterol-containing membranes. These results indicate that the oligomeric nature of channel assembly is not strictly fixed. The gating transitions occur through conformational changes of a defined oligomer. Also, no flop of the *trans* ergosterol was detected. These results strongly suggest that AM3 forms barrel-stave type channels^25–27^ at low concentrations. At this concentration, no pores were detected by AFM, since the low density of the channel on the membrane does not allow channel images to be captured (only a few channel should exist on the membrane area of <1000 μm^2^ in the single-channel current recordings). However, at the mid-concentration range (0.4 μM), slight membrane deformation was detected even though the pore could not be identified.

At a high AM3 concentration (2.0 μM), it was surprising to detect the jumbo channels, which were resolved as discrete single channel currents superimposed on macroscopic currents of the smaller conductance channels observed at lower concentrations. The single-channel conductance showed substantial variability (20–50 nS), and the estimated pore diameter based on the conductance was 2.6–4.0 nm. In parallel, AFM revealed a pore approximately 10 nm in size, even though the narrowest diameter inside of the pore remains elusive. These range estimates of the pore size from different experimental methods are consistent given each method has its inherent limitation. This size corresponds to several tens of AM3 molecules self-assembled around the pore with ergosterol. The most unexpected effect of AM3 was the formation of a wrinkle phase on the membrane. The width of the wrinkle overwhelmed the molecular size of AM3, and the thicker membrane phase of 5.5 nm was induced in the flat surface with an ordinary membrane thickness of 4.7 nm. This phase change was also supported by the attenuated diffusion of the lipid at high AM3 concentration (Fig. S3).

In this high concentration range, we found, for the first time, the *trans*-side effect of ergosterol, in which the flip-flop of ergosterol along the concentration gradient between the outer and inner leaflets was facilitated only after the formation of the jumbo channels. These results indicate that a continuity was established between the outer and inner leaflet of the membrane. This feature conforms closely to the feature of toroidal pore, and in the absence of the formal definition of the toroidal pore, we conclude that the jumbo channel forms a toroidal-type pore^27, 30–32^.

**Figure 6:**
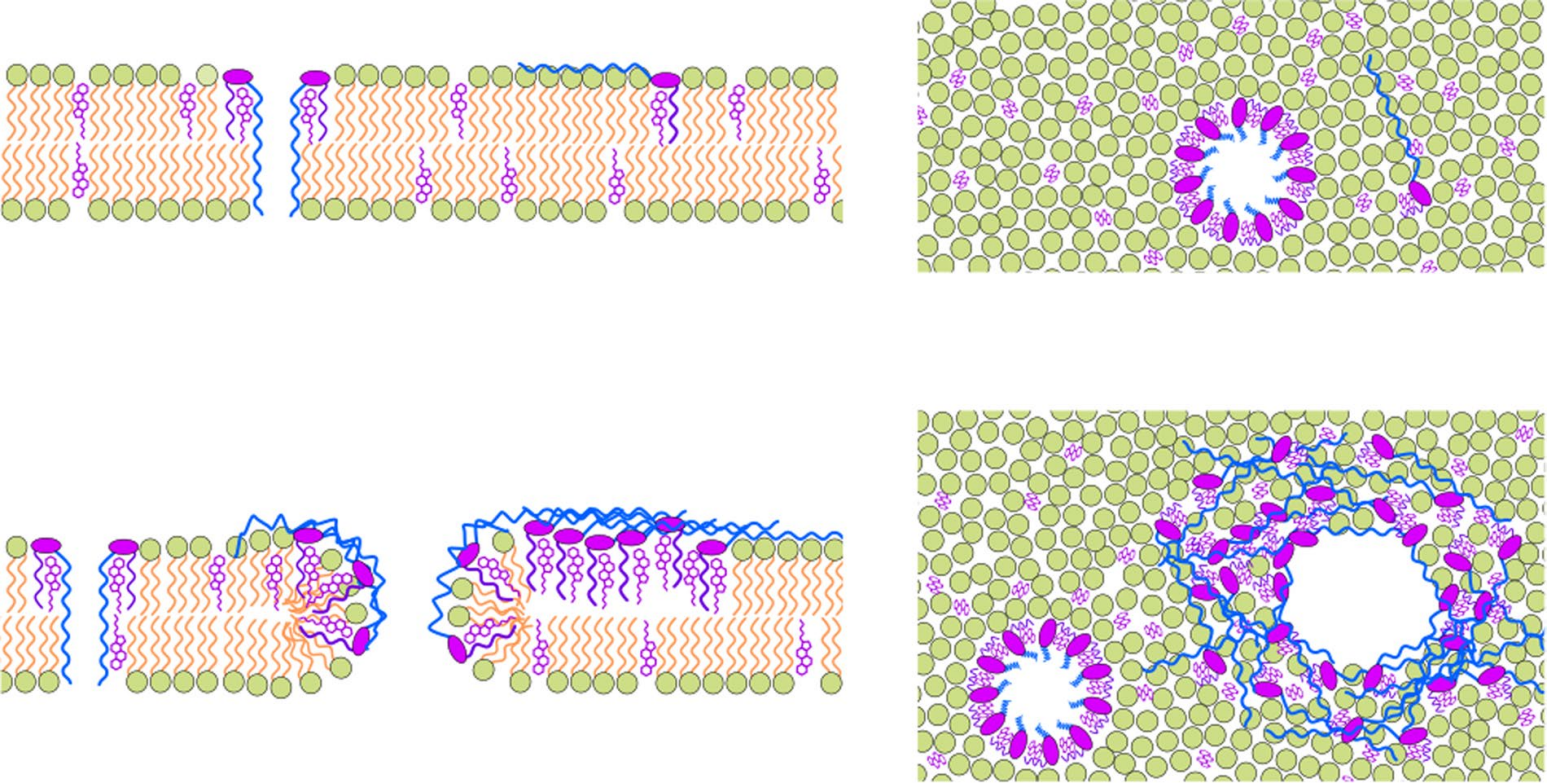
Schematic representation of AM3 assembly in the membrane at different AM3 concentrations. Side view (left column) and top view (right column) of an AM3-embedded membrane. With the hydrophobic chain anchored in the sterol-containing membrane, AM3 takes on either the η or Γ shape. At a low AM3 concentration (upper), the η-shaped AM3 with a long hydroxyl chain spans the membrane and forms a barrel-stave pore through self-assembly. At a high concentration (lower), the Γ-shaped AM3, with its OH chain located at the membrane interface, induces membrane phase separation similar to that observed in the raft, in which jumbo pores are generated. The jumbo channels facilitate the flip-flop of ergosterol, sharing properties with the toroidal-type pore.

The polymorphic channel activity and the membrane phase-inducing activity are related to the structural features of the AM3 molecule (Fig. 1A). The linear molecular structure of AM3, in contrast to the cyclic structure of other polyenes, allows AM3 to attain structural variety. A previous study assumed that the rigid tetrahydropyran moiety of AM3 closely interacts with the OH of ergosterol stereo-specifically^33^. This rigid part forms the structural core, and the shorter hydrophobic chain is inserted into the membrane, which anchors AM3 to the sterol-containing membrane (Fig. 6). The membrane anchoring secures AM3 more intimately interacted with the membrane by taking either of two shapes. When the long amphipathic tail lies at the membrane surface, AM3 takes the Γ shape, whereas the long chain inserts into the membrane towards the *trans* leaflet to form the η-shape.

We present here a hypothetical mechanism of the formation of AM3 channels in the membrane. Both molecular shapes likely appear at all concentration ranges. At the low-concentration range, the barrel-stave channel is formed by the η-shaped AM3, and the pore interior is hydrophilically lined. At the high-concentration range, phase separation of the membrane and jumbo pore formation occur. The membrane thickness is increased substantially in the wrinkle phase, and the Γ-shaped AM3, which interacts with sterol, should contribute to this phase change. This type of interaction with the membrane is distinct from the previously proposed carpet model, in which toxic peptides spread onto the membrane surface by interacting with the lipid head groups^32, 34^. Rather than inducing a disordered membrane in the carpet model, AM3 renders the membrane phase separated, and stable jumbo channels are formed. The intimate interaction between AM3 and sterol reminds us of the interaction between sphingomyelin and cholesterol in membrane rafts^35–37^. In the raft and the AM3-induced wrinkle phase, common physicochemical principles are likely underlying. In the raft-like phase, the membrane is deformed to generate pores, by which ergosterols are freely transferred between the leaflets via an established continuity. This type of pore is distinct from the barrel-stave type. Additionally, the pore in our hypothetical model (shown in a schematic in Fig. 6) shares features of the toroidal pore, accounting for the structural and functional observations described herein. Although the definition of the toroidal pore has not reached a consensus and this concept has been applied exclusively to peptides and proteins, the polyene macrolide AM3 is possibly categorized as a toroidal pore because it induces the flip-flop of membrane constituents.

The channel activity and sterol dependence of polyenes (nystatin and amphotericin) have been exploited as an experimental tool for electrophysiological measurements. In the perforated patch method, patch membranes are permeabilized, and the pipette solution in the patch pipette and intracellular space are electrically connected, even without membrane breakage, which prevents the loss of macromolecules in the cytosol towards the pipette solution^38–40^. Additionally, in planar lipid bilayer experiments, nystatin and sterol in the liposomes facilitated the fusing of channel-reconstituted liposomes to the lipid bilayer^41, 42^. These uses of polyenes suggest that the unique features of AM3, including the phase-inducing activity, can be exploited to manipulate membrane properties in an unprecedented manner.

The unusual concentration dependency is supposed to be relevant to the toxic action of AM3. Cells, having sterols in the membrane, closely located to dinoflgellates are immediately disrupted upon exposure to high concentration AM3. On the other hand, remote cells having exposed to low AM3 loose the membrane potential, but they may survive or even activated depending on their cellular properties. These contrast functional outcomes at low and high concentrations are predicted to undergo *in vivo*, which is unique for AM3. This feature may contribute development of anti-fungal compounds having less side effects^42–44^.

## Methods

### Materials

POPC, ergosterol, and epicholesterol were obtained from Avanti Polar Lipids (Alabaster, AL), Tokyo Kasei (Tokyo, Japan), and Cambridge Isotope Laboratories, Inc. (Tewksbury, MA), respectively. Cholesterol and other chemicals were purchased from Nacalai Tesque (Kyoto, Japan). AM3 was isolated from a culture of the dinoflagellate *A. klebsii* as previously described^4^.

### Contact bubble bilayer method

The electrophysiological properties of the AM3 pores formed on the lipid bilayer were analyzed by the CBB method^11^. Experiments were performed in a small glass chamber filled with hexadecane (150 μL) on the stage of an inverted microscope (IX73, Olympus, Tokyo, Japan). Two glass pipettes (OD/ID; 1.50/1.05 mm, Hilgenberg GmbH, Malsfeld, Germany) were filled with 1 M KCl solution containing 2 mg/mL liposomes (90 mol% POPC, 10 mol% sterols). The liposome solution was swelled at the tip of each pipette (diameter, 30 μm) in hexadecane to form small water in oil (w/o) bubbles coated with a lipid monolayer. Two bubbles were placed in contact with each other by pipette manipulation, forming the CBB at the contacting face. Ag/AgCl wire electrodes (E255, Warner Instruments, Hamden, CT) were placed inside of the bubble-forming pipettes, and the ionic current across the CBB was measured under membrane voltage-clamped conditions using a patch clamp amplifier (EPC800USB, HEKA, Lambrecht/Pfalz, Germany). AM3 molecules were added to liposome solutions connected to the reference electrode; this side was defined as the *cis* side. The current signals were filtered (1 kHz cutoff frequency) and sampled at 5 kHz by an A/D converter (Digidata 1550 A, Molecular Devices) and stored using pCLAMP software (Molecular Devices, Sunnyvale, CA).

Corrections for the electrode potential and the liquid-junction potential for the bi-ionic solutions were performed as follows. For the electrode potential, the offset was adjusted before the membrane formation and immediately after the membrane break (two bubbles were fused). The liquid-junction potential was corrected by the calculation of the value in the given ionic compositions^45^.

The reversal potential was evaluated from the permeability ratio, which was calculated using the Goldman-Hodgkin-Katz equation.

### Estimation of the pore radius

For large-conductance channels, the geometrical size of the pore can be estimated by assuming the macroscopic regime of current flow^21^. Therefore, the following equation was applied.

1 $$\begin{array}{c}{R}_{pore}=\frac{\rho \,l}{A}=\frac{\rho \,l}{\pi \,{r}^{2}}=\frac{l}{\kappa \pi \,{r}^{2}}=\frac{1}{{g}_{pore}}\end{array}$$

$$\begin{array}{c}\quad \,\,\,r=\sqrt{\frac{{g}_{pore}\,l}{\kappa \pi }}\end{array}$$

Here, κ is 0.11132S/cm (at 1 M KCl, 25 °C), *l* is assumed to be 30 Å, and the measured *g*_pore_ was 1.7 nS (at 1 M KCl, 25 °C); thus, *r* was 3.8 Å.

### AFM imaging of AM3 pores in supported lipid bilayers

A micellar solution of 0.5 mg/mL POPC with 10% sterols in DDM-containing buffer (10 mM HEPES, pH 7.5, 200 mM KCl, and 0.06% DDM) was diluted 10 times with the buffer without DDM to form liposomes. The liposome solution was placed on a freshly cleaved mica surface. The bilayers were absorbed on the mica surface, resulting in partially formed supported lipid bilayers. After a 15-min incubation, the floating liposomes and detergent were rinsed off with buffer (10 mM HEPES, pH 7.5 and 300 mM KCl). The solution was then replaced with recording solution containing AM3 (10 mM HEPES, pH 7.5, 300 mM KCl, and 4.5 μM AM3). Images were taken with a Cypher (Oxford Instruments) using AC mode at 28 °C. BL-AC40TS (OLYMPUS) cantilevers were used and had a tip radius and spring constant of approximately 7 nm and 0.1 N/m, respectively. Image analysis was performed using Gwyddion software (Department of Nanometrology, Czech Metrology Institute, Czech).

## Electronic supplementary material

Supplementary Information
