# Supplementary material for: Channel Formation and Membrane Deformation via Sterol-Aided Polymorphism of Amphidinol 3
Source: Sci Rep. 2017 Sep 7;7:10782. doi: 10.1038/s41598-017-11135-x (PMC5589915; doi:10.1038/s41598-017-11135-x)
Supplement: Supplementary file 1 — Supplementary Information [file 41598_2017_11135_MOESM1_ESM.pdf]

Supplementary Information

Channel Formation and Membrane Deformation via Sterol-Aided Polymorphism of Amphidinol 3

Masayuki Iwamoto, Ayumi Sumino, Eri Shimada, Masanao Kinoshita, Nobuaki Matsumori and Shigetoshi Oiki

[1]Department of Molecular Physiology and Biophysics, University of Fukui, 910-1193, Japan
[2]PRESTO, Japan Science and Technology Agency (JST), Saitama 332-0012, Japan
[3]Department of Chemistry, Faculty and Graduate School of Sciences, Kyushu University, 819-0395, Japan
[4]Present address: High-speed AFM for Biological Application Unit, Institute for Frontier Science Initiative, Kanazawa University, Kanazawa 920-1192, Japan; Bio-AFM frontier Research Center, Kanazawa University, Kanazawa 920-1192

Single-channel current of AM3 in a cholesterol-containing membrane

The single-channel current recordings revealed various types of channel activities having variable conductance and gating (Fig. S1A). The single-channel current-voltage curves varied substantially in the cholesterol-containing membrane.

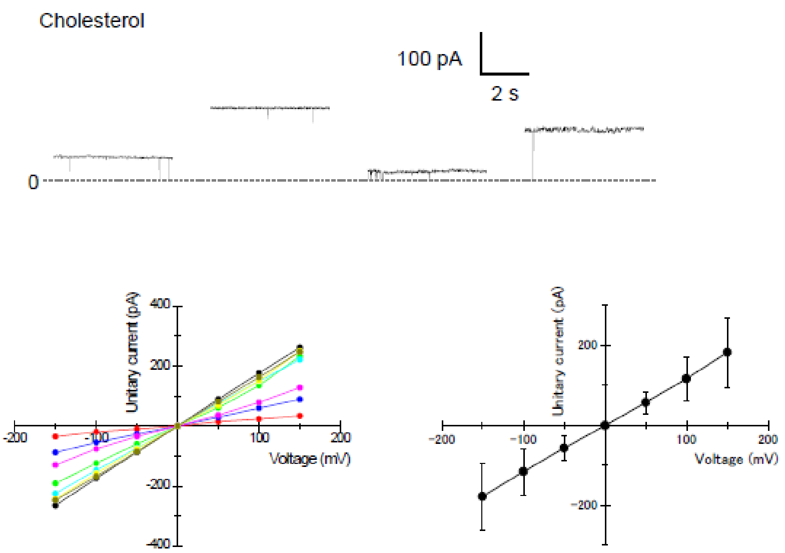

Fig. S1. Single-channel current of AM3 in a cholesterol-containing membrane.

AFM images of sterol-containing membrane without and with AM3

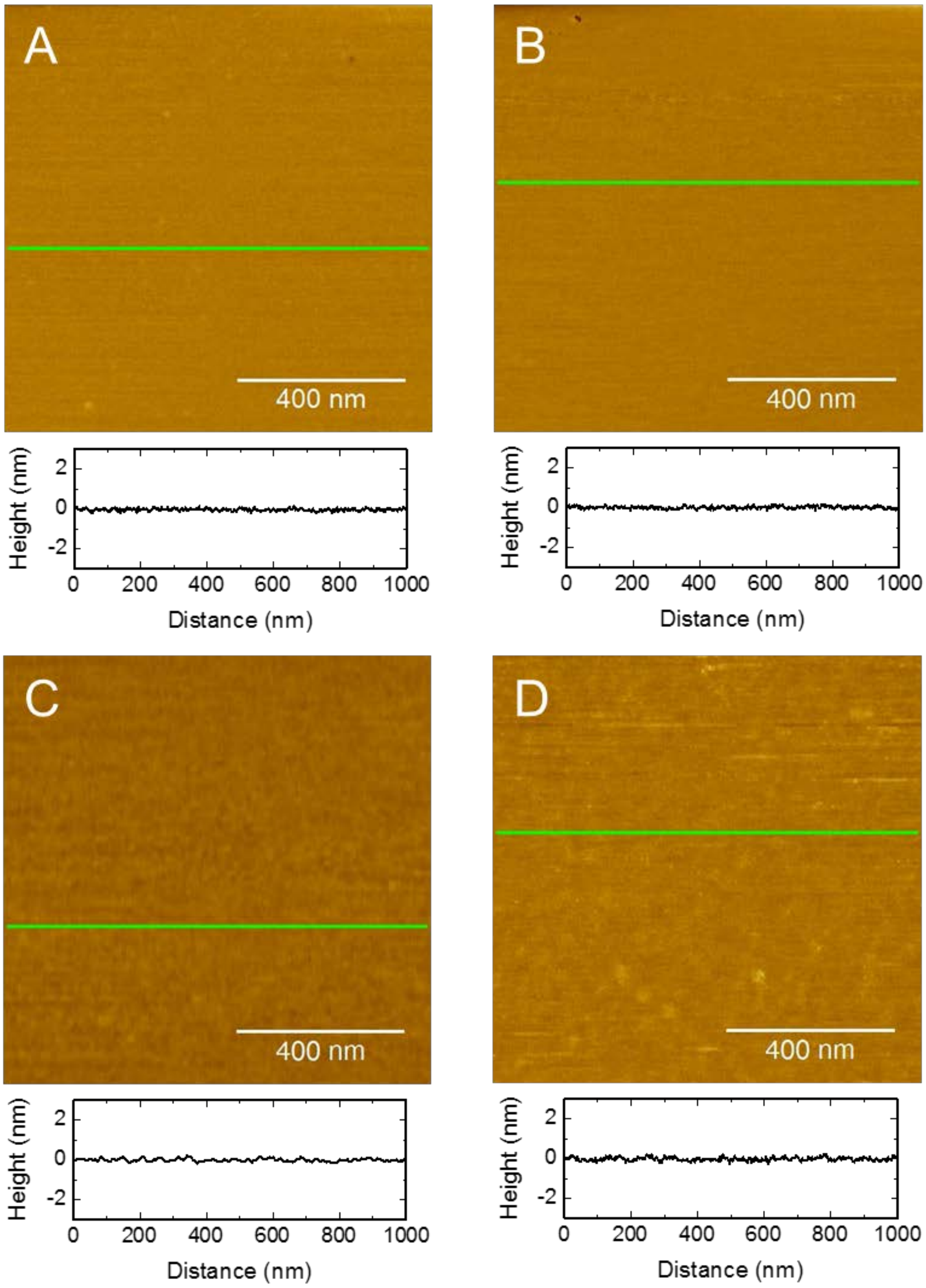

Figure S2. AFM images of sterol-containing POPC bilayers without (A, B) and with 0.4 μM AM3 (C, D). The membranes contain 10 % of sterols (cholesterol for A and C, ergosterol for B and D). The height profiles along the green solid lines are shown below the image. The height of the bilayer surfaces was set to 0 nm. Images were taken in a solution containing 300 mM KCl and 10 mM HEPES (pH 7.5).

GUV experiment

GUV preparation

GUVs of ternary mixtures of lipids were prepared by electroformation (Angelova, M.I., and Dimitrov D.S. *Faraday Discuss. Chem. Soc. 1986, 81,303*). In brief, an aliquot (7 µL) of a POPC/cholesterol (9:1) solution (1 mg/mL, $CHCl_3$-MeOH) containing 0.002 mol% 594neg-DOPC (Kinoshita M. et al *J. Cell Biol. 2017, 216, 1183*) was spread on the electrode surface (platinum wires, Φ = 100 µm), and the remaining solvent was removed under vacuum for 24 h. Two electrodes coated with the thin lipid film were attached on both sides of a square-shaped rubber seal (1 mm thick, with a square window of 15 × 15 mm, containing 0.4 mL Milli-Q water), and then were sandwiched by two cover glasses (24 × 60 mm, 0.12- to 0.17-mm thickness; Matsunami). This chamber was placed on a temperature-controlled sample stage (Tokai Hit) on an inverted confocal fluorescence microscope, and then a low-frequency (10 Hz) sinusoidal current (10 V_{pp}) was applied, using an Agilent Technologies function generator (model 33120A) at 55°C for 60 min (the temperature was calibrated by a K-type thermocouple sensor, AD-5602A; Sanyo Industries). The GUVs formed in this chamber were maintained at 25°C for 15 min. To the GUV suspension, an AM3 aqueous solution was added. The final concentrations of AM3 were 4.5 and 9.0 µM.

Confocal fluorescence observations and FCS measurements

Confocal laser-scanning fluorescence microscopy was performed with an FV1000-D microscope (IX81; Olympus), using an objective lens with a long working distance (LUCPLFLN 60×, NA 0.7). The image was obtained by the acquisition software FV10-ASW4.2. The contrast and the brightness of the obtained images were adjusted with Adobe Photoshop.

FCS measurements of GUV surfaces were performed at 28°C with the same microscope used to obtain the confocal fluorescence images using an oil-immersion apochromat objective lens (Olympus PLAPON60XO, 60×, NA 1.4). The excitation laser light (594 nm) was focused on the flat top part of the GUV membrane (the membrane opposite from the coverslip side). The confocal geometry was ensured by obtaining the confocal images before FCS measurements. The diffusion coefficients were obtained as previously described (Kinoshita M. et al *J. Cell Biol. 2017, 216, 1183*).

A

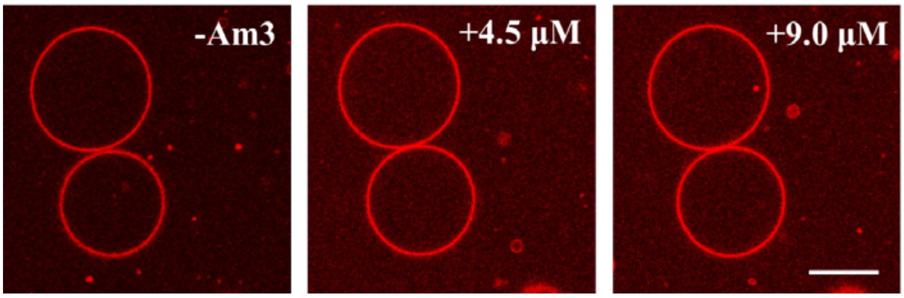

B

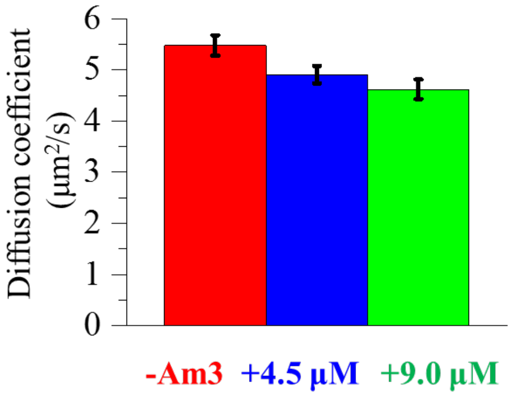

Fig. S3. Confocal fluorescent microscopy images of GUVs stained with fluorescence-labeled DOPC in the absence and presence of AM3 (A) and diffusion coefficients of the fluorescence-labelled PC without and with AM3 (B). GUVs were composed of POPC and cholesterol (9:1). Obvious phase separation was not resolved under the fluorescence microscopy (A), but the diffusion of PC was gradually attenuated as the AM3 concentration increased (B).
